# Continuous Monitoring Biosensing Mediated by Single-Molecule Plasmon-Enhanced Fluorescence in Complex Matrices

**DOI:** 10.1021/acsnano.3c12428

**Published:** 2024-02-09

**Authors:** Vincenzo Lamberti, Mathias Dolci, Peter Zijlstra

**Affiliations:** Eindhoven University of Technology, Department of Applied Physics and Science Education and Institute for Complex Molecular Systems, 5600 MB Eindhoven, The Netherlands

**Keywords:** plasmon sensing, nanoparticles, single-molecule, fluorescence, continuous monitoring

## Abstract

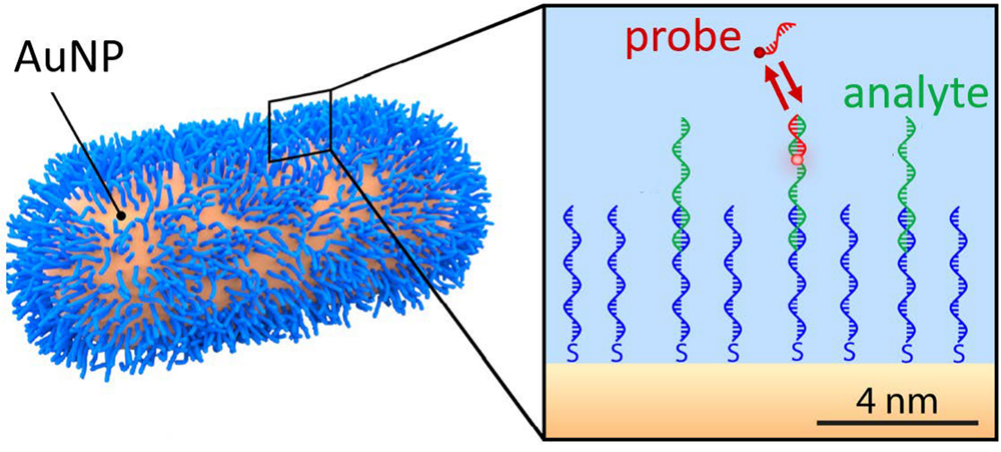

Continuous detection of critical markers directly at the point of interest and in undiluted biological fluids represents the next fundamental step in biosensing. The goal of realizing such a platform is utterly challenging because it requires a reversible biosensor that enables the tracking of pico- to nanomolar molecular concentrations over long time spans in a compact device. Here we describe a sensing method based on plasmon-enhanced fluorescence capable of single-molecule detection of unlabeled analyte by employing biofunctionalized gold nanoparticles. The very strong plasmon-enhanced fluorescence signals allow for single-molecule sensing in unaltered biological media, while the use of low-affinity interactions ensures the continuous tracking of increasing and decreasing analyte concentrations with picomolar sensitivity. We demonstrate the use of a sandwich assay for a DNA cancer marker with a limit of detection of picomolar and a time response of 10 min. The enhanced single-molecule signals will allow for miniaturization into a small and cheap platform with multiplexing capability for application in point-of-care diagnostics, monitoring of industrial processes, and safe keeping of the environment.

## Introduction

The ability to detect and track biomolecules in situ has been crucial in our understanding of the dynamics of natural processes. In addition, biomolecular sensing has greatly impacted diagnostics and treatment,^1^ industrial process optimization,^2,3^ and environmental monitoring.^4,5^ Laboratory-based biosensing technologies are available for a plethora of markers yet present long sample-to-data time scales and therefore a high cost per data point. An ideal biosensor would enable continuous monitoring by quantifying analyte concentrations directly in biological media with high sensitivity, without requiring washing steps. Commercially available continuous biosensors are limited to electrochemical detection of highly concentrated markers such as glucose,^6^ lactate,^7^ and cortisol.^8^ Such devices are based on enzymatic technology and, therefore, work specifically for biomarkers that are enzymatic substrates. This makes detection not easily generalized to other analytes, which therefore requires a different method rooted in affinity-based detection.

Developing a continuous monitoring technology reliant on affinity-based detection requires a reversible binding mechanism between the analyte and capture probe. This can be accomplished by using low-affinity capture probes that bind the analyte for (typically) less than a minute before unbinding.^9^ Such reversible and short-lived interactions provide the ability to track increasing as well as decreasing biomarker concentrations in real-time, without requiring repeated chemical regeneration.^10,11^ Continuous monitoring using low-affinity interactions has been reported via aptamer-based sensors,^12−16^ but ensemble-averaged readout using electrochemistry limits the sensitivity to nano- to micromolar concentrations. To overcome such limitations single-molecule resolution^17−20^ is needed since it resolves individual binding events even if the time-averaged occupancy of the capture probes is low.

Recently, biosensing by particle mobility has been proposed^21,22^ wherein the Brownian motion of micron-sized particles is modulated by single-molecule interactions with a planar surface. These reversible interactions can be continuously monitored optically, while the single-molecule sensitivity provides access to picomolar concentrations. However, micrometer-sized probes are susceptible to nonspecific interactions due to their large and heterogeneous surface, prohibiting their use in unfiltered biological fluids such as serum or plasma. Fluorescent probes such as organic fluorophores, on the other hand, are small and compatible with biological fluids.^23−26^ In diagnostics, single-molecule fluorescence has proven capable of detecting both RNA^27^ and small proteins^28−30^ achieving high specificity and femtomolar limit-of-detection. However, the limited optical signal of single organic fluorophores is easily overcome by spurious background fluorescence from, e.g., aromatic amino acids in serum proteins. Current assays therefore require several washing steps after analyte binding to reduce this background and are therefore not suitable for continuous monitoring.

Plasmonic nanoparticles are revolutionizing the field because they can enhance a dye’s fluorescence intensity >10^3^-fold resulting in a vastly improved signal-to-background ratio.^31−33^ These have been employed to probe single biomolecules in highly concentrated samples^23,34^ and to improve the overall brightness of single fluorophores.^35−37^ Plasmon-enhanced fluorescence has recently been applied to the field of diagnostics,^38,39^ where multiple assay designs were used to detect analyte in point-of-care devices. The strong plasmon-enhanced signals generated by a dimeric nanogap antenna enabled miniaturization of the optical setup to a point-of-care device, thereby highlighting the promise of the technology for biosensing. However, continuous monitoring was not possible due to the poor accessibility of the nanogap and the need for washing steps in the assay.

Here we demonstrate a biosensor that is capable of continuous monitoring of analyte concentrations directly in undiluted blood serum without the requirement of washing steps. Hundreds of individual gold nanorods are probed in parallel, acting as an antenna to strongly enhance the fluorescence of detection probes. We employ a low-affinity sandwich assay wherein the unlabeled analyte reversibly binds to capture probes on the particles. The presence of bound analyte is then quantified by the repeated binding of low-affinity detection probes. The advantage of this approach is twofold: on the one hand, the short-lived interactions enable continuous monitoring biosensing at pico- to nanomolar concentrations. On the other hand, the use of PEF overcomes background signals of complex media and enables detection directly in full blood serum. Using this platform, we demonstrate the monitoring of a tumor DNA marker with picomolar limit of detection (LOD) and a time-response of 10 min. The use of single-molecule PEF biosensors will enable the continuous monitoring of biomolecules ranging from nucleic acids to proteins and peptides in point-of-care devices for biomedical, industrial, and environmental applications.

## Results and Discussion

**Figure 1:**
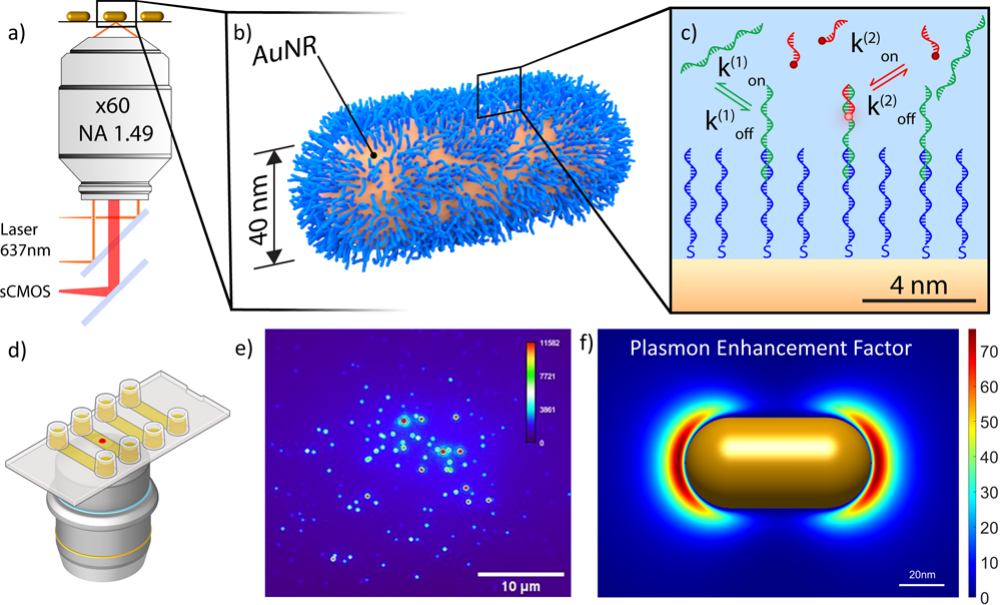
Sketch of the experimental setup. (a) Schematic of the total internal reflection fluorescence (TIRF) microscope comprising an oil immersion objective, a 637 nm laser, and a sCMOS camera. (b) Cartoon of a gold particle decorated with a high density of capture probes (in blue). (c) Schematic of the reversible sandwich assay including low-affinity ssDNA capture probes (blue), the analyte sequence (green), and low-affinity detection probes labeled with Atto655 (red). (d) Cartoon of the used multichannel flowcell. (e) Example of a typical wide-field fluorescence image, where each diffraction-limited spot indicates the presence of a nanoparticle. (f) Numerical simulation of the total fluorescence enhancement for a 40 nm diameter gold nanorod with a longitudinal plasmon resonance of 640 nm. Simulated for an excitation wavelength of 637 nm polarized along the long axis of the nanorod, and an emission wavelength of 680 nm representing ATTO655, assumed to be freely rotating.

The detection principle is based on a single-molecule fluorescence sandwich assay that is performed on the surface of single nanoparticles that enhance the fluorescence intensity. The sensor consists of immobilized gold nanorods (AuNRs) on a glass substrate at a low particle density in an objective-based total internal reflection microscope (Figure 1a). The gold particles are decorated with single-stranded DNA as a capture probe (Figure 1b, sequences can be found in Supporting Information (SI)). The capture probe has a short complementary region with the unlabeled analyte, that therefore binds weakly on time scales of approximately 1 s. Here the presence of analyte molecules on the particle is probed using a fluorescently labeled (ATTO655) detection probe (Figure 1c) in a similar manner to DNA Point Accumulation for Imaging Nanoscale Topography (DNA-PAINT).^40^ The substrate with functionalized particles is inserted into a multichannel fluidic cell (Figure 1d). Figure 1e shows a typical fluorescence image obtained in the absence of detection probes, where each diffraction-limited spot is due to the one-photon photoluminescence of a single gold nanorod.

The temporary formation of the complex between the analyte and detection probe brings the dye into the vicinity of the plasmonic nanoparticle, thereby generating PEF. Figure 1f reports the numerically simulated total plasmon enhancement factor for the used ATTO655 dye in the proximity of a AuNR with a plasmon resonance of 640 nm. PEF is observed only near the particle’s surface, resulting in a strong enhancement of the signal of bound detection probes. Any nonspecific interactions of the detection probe and/or other fluorescent species with the glass substrate result in substantially lower signals and are hence not detected. Typical measurements allow probing a few hundred NPs simultaneously, as shown in Figure 1e, providing high statistics for precise concentration measurements.

### Biosensing and Analysis Method

**Figure 2:**
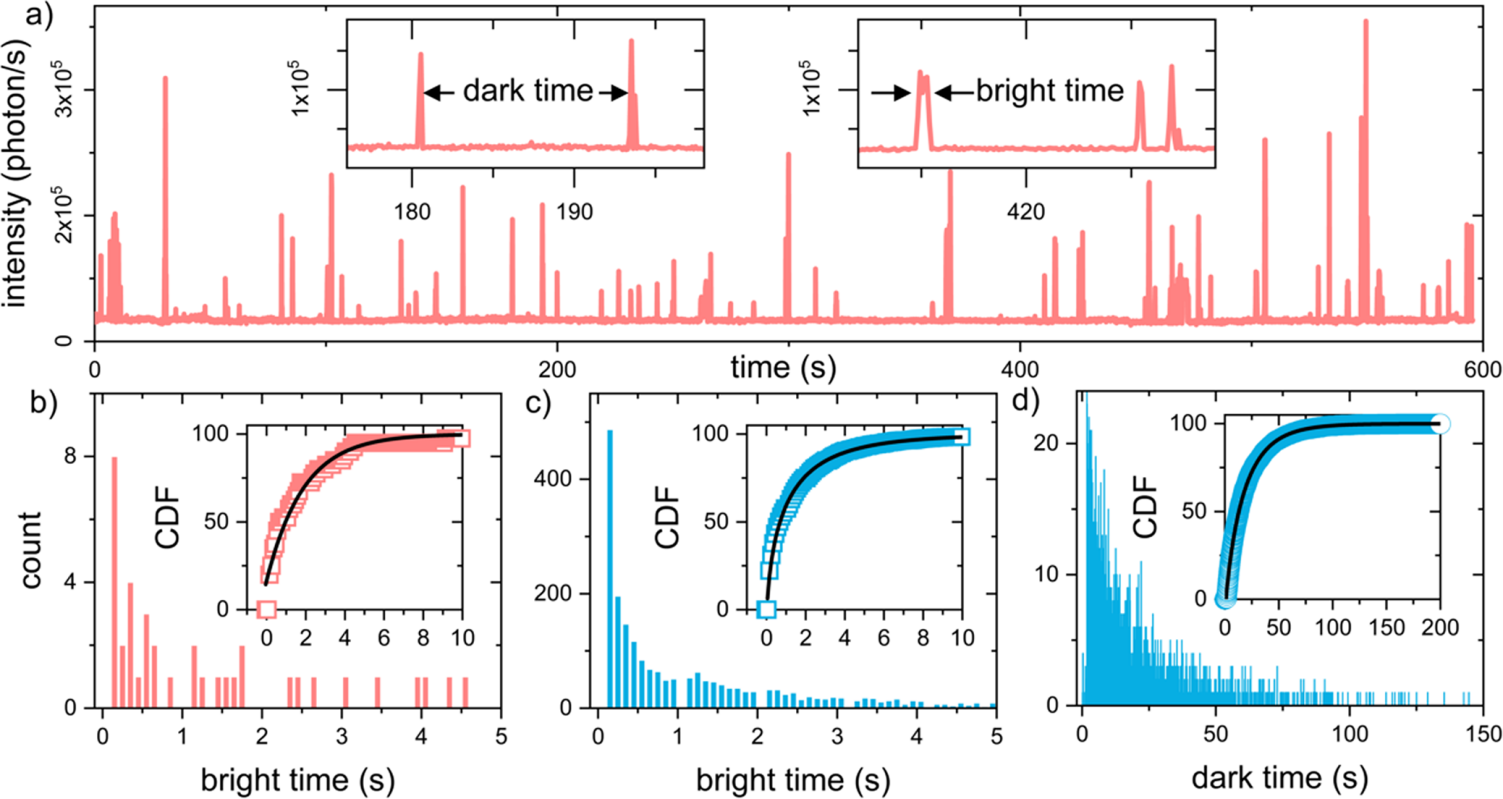
Typical time trace and analysis process. (a) Example TT of a single gold nanorod showing fluorescence bursts due to detection probes (10 nM) that reversibly bind to an analyte (300 pM) in a sandwich assay format. The insets highlight the concepts of dark time between consecutive events and bright time as event duration. (b) Distribution of bright times obtained from the TT in (a). The inset shows the CDF of the same data set with a single exponential fit (fit parameter τ_*b*_ = 1.18 s). (c) Distribution of bright times for all 88 particles in a typical FoV. The inset shows the CDF of the same data set fitted with a stretched exponential fit with fit parameters τ_*b*_ = 1.03 s and stretching factor γ = 0.62. (d) Distribution of dark times for the same particles, the CDF is fitted with a stretched exponential with fit parameters τ_*d*_ = 17.28 s and stretching factor γ = 0.86). Bins size is fixed to 100 ms as exposure time.

Here, we present the experimental and analysis methods following common approaches of single-molecule localization microscopy as pioneered by the DNA-PAINT community. Within a flowcell, mixtures containing analyte and detection probe are flown onto the sensing surface. The sample is imaged on a fluorescence microscope, where the sensor activity is captured by a sCMOS camera. The frequency of detection-probe binding is recorded in an image sequence of 10 min and processed as follows. First, nanoparticles are identified in a drift-corrected image sequence, resulting in a few hundred AuNRs per data set. Second, for each localized nanoparticle, the point spread function is fitted with a 2D Gaussian function, generating a fluorescence time-trace (TT) of the volume under the 2D Gaussian. To ensure only monomer nanoantennas were further processed, correlative dark-field microscopy is performed on a typical sample to obtain scattering spectra alongside fluorescence TTs. Typically, 5–10% of spots contain clustered nanoparticles represented by a non-Lorentian-shaped scattering spectrum. Such clusters also result in an unstable photoluminescence baseline in the TTs, possibly due to dynamic changes in the gap size. On the contrary, TTs with a stable baseline, as shown in Figure 2a, are solely identified as single AuNRs with a narrow Lorentian-shaped spectrum (from 150 to 250 meV) and longitudinal plasmon resonance around the expected 650 nm. In typical data sets, only fluorescence microscopy measurements were performed, and unstable TTs are assumed to be clustered particles, hence discarded from further analysis. More details on the identification of single particles can be found in the SI.

In the extracted TTs the single-molecule interactions of detection probes binding to a captured analyte are identified by fluorescent bursts. Each fluorescence burst has a different peak intensity because stochastic binding events occur at different locations on the particle, leading to different enhancement factors. Using a Matlab app, such events were detected by thresholding the TTs followed by quantification of their duration and waiting time, hereafter referred to as the bright and dark times. A detailed discussion of this process can be found in the SI. A major advantage of this single-molecule sensing principle is the digital nature of the signals: binding events can be simply detected by thresholding without suffering from signal drift due to mechanical movement or temperature fluctuations.

For each field-of-view (FoV), every event is characterized by peak intensity, bright time, and dark time. For each single particle, the cumulative distribution function (CDF) of bright and dark times is fitted with a single-exponential. The obtained typical decay times are defined as characteristic times τ_*b*_ and τ_*d*_ for each particle (Figure 2b). Similarly, the distributions of dark and bright times collected from all particles in the FoV are fitted with a stretched exponential function (Figure 2c–d). The bin size is fixed to 100 ms as the experimental camera exposure time. The dynamics of single-molecule interactions between the detection probes and bound analyte define τ_*b*_ and τ_*d*_ for every single particle. τ_*b*_ directly relates to the dissociation rate of the detection probe, which can be controlled by the length of complementary bases and buffer conditions. Given typical molecular dynamics^27^ and comparing enhanced and nonenhanced fluorescence intensities, a kinetic finger-printing filter is applied to remove short and low-intensity detections arising from noise or unspecific binding (see SI for a description of the event filtering).

### Sensor Response

Here, we present the sensor’s digital response as well as the modeled dose–response curve following Langmuir–Hill kinetics. The digital signals of the sensor can be quantified based on the average event frequency *f*_*E*_ or characteristic τ_*d*_ extracted from the CDF of all events in the FoV. Using an adsorption model for a sandwich design dictated by Langmuir kinetics, the event frequency can be determined given the equilibrium constants of capture probe–analyte and analyte–detection probe interactions:
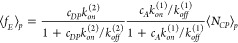
1where  is the average event frequency per particle, *c*_*DP*_ and *c*_*A*_ are detection probe and analyte concentrations respectively, *k*^(1)^ and *k*^(2)^ indicate the rate constants for capture–analyte and analyte–detection probe interactions respectively, and  is the average estimated number of capture probes per particle. As a result, the frequency of events in a TT is proportional to the analyte concentration when the detection probe concentration is constant. To fit the measured data, eq 1 is modified by accounting for the Hill coefficient, while all prefactors are absorbed into the constant α. This gives
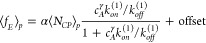
2where *k*_*on*_^(1)^/*k*_*off*_^(1)^ are fit parameters while *c*_*DP*_ is fixed at the experimental value of 10 nM. Details on the derivation of this equation can be found in the SI.

Hundreds of single AuNRs can be processed simultaneously to achieve a high statistical precision. Due to the functionalization process of AuNRs, the number of capture probes per particle  is Poisson-distributed across the nanoparticles. In eq 2 the event frequency is assumed proportional to  whose value was measured with quantitative PAINT following the methods illustrated by Horacek et al.,^41^ resulting in . Consequently, the expected average event frequency per particle is proportional to the number of captured analytes and is necessarily Poisson distributed. Hereafter, all values are reported as a mean over all particles in the FoV, while the sensor precision is associated with the standard error (SE) of the mean.

### Dose Response in Buffer

**Figure 3:**
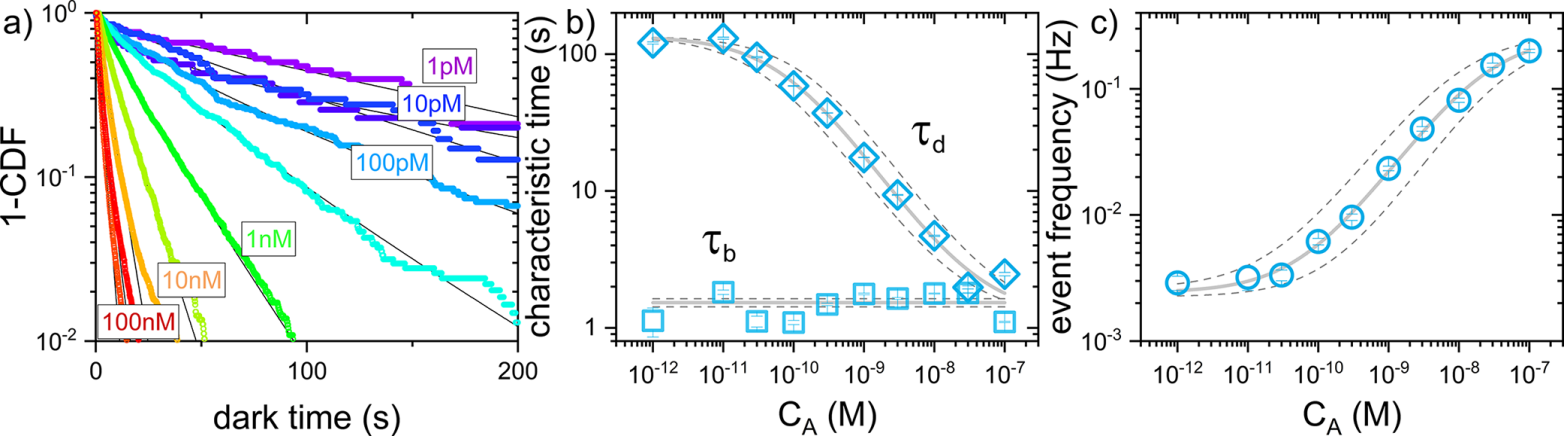
Sensor response for model assay in sensing buffer. (a) Cumulative distribution function of measured dark times for different analyte concentrations. Solid lines are stretched exponential fits (stretching factor γ varying from 0.62 to 0.94). (b) Average τ_*d*_ and τ_*b*_ for different analyte concentration. τ_*d*_ are fitted according to eq 2 with a minus sign. Fit parameters are Hill coefficient 0.76, α = 0.24, offset = 135 and *k*_*on*_^(1)^/*k*_*off*_^(1)^ = 4.73 × 10^7^ M^–1^. τ_*d*_ are fitted with constant value = 1.52. Solid and dashed lines are fitting curves plus/minus errors in the fitting parameters. (c) DR curve plotted for the mean event frequency per particle. Average values across 3 repeats. Error bars indicate the average standard error of the mean. Data are fitted according to eq 2. Fit parameters are Hill coefficient 0.78, α = 0.2, offset = 2.4 × 10^–3^ and *k*_*on*_^(1)^/*k*_*off*_^(1)^ = 9.9 × 10^5^ M^–1^. Solid and dashed lines are fitting curves plus/minus errors in the fitting parameters.

First, we demonstrate the sensor response in a clean buffered solution (see the Methods section for buffer compositions). This is done for a model DNA assay that exhibits a 12 nt complementarity between the capture probe and the analyte, and a 9 nt complementarity between the analyte and the detection probe (see SI for the sequences). This results in fluorescence bursts whose durations are largely dictated by the 9 nt complementary region, leading to average burst durations of approximately 1 s as shown in Figure 2. The detailed sensor response as a function of the analyte concentration is shown in Figure 3.

The response experiments are performed on a single sensing substrate, progressively increasing the analyte concentration and recording the activity. Fluids in the measuring chamber are replaced by injecting the next measuring condition in a volume 4–5-fold the flow cell’s capacity. The distribution of dark times across the whole FoV is shown in Figure 3a for different analyte concentrations. As expected, the dark times are exponentially distributed and shorten as the concentration of analyte increases. Exponential fits to the distributions yield the characteristic τ_*b*_ and τ_*d*_ that are shown in Figure 3b. Here the τ_*b*_ (blue squares) remains constant for changing analyte concentrations since the detection probe–analyte dissociation rate is not dependent on the analyte concentration. In contrast, the values for τ_*d*_ decrease with increasing analyte concentration due to a higher event frequency as shown in Figure 3c. However, too high concentrations of the analyte will result in temporally overlapping events where two or more detection probes are bound to one NR at the same time. In this regime, the sensor response saturates since double events cannot be distinguished. At low analyte concentrations, the sensor response is dominated by nonspecific signals and noise in the time traces that are picked up by the detection algorithm. As a result, for this model DNA assay, the sensor exhibits an LOD of 5 pM with 3 orders of magnitude of dynamic range. Fits of eq 2 to the dose–response curve give typical Hill coefficients γ = 0.93 ± 0.1. This indicates that the binding of additional analyte is inhibited by analyte already bound, possibly due to electrostatic repulsion or steric effects. A full discussion on the sensors’ kinetics and response parameters, such as LOD and LOQ, can be found in the SI section “Sensor analytical response parameters”.

### Response in Complex Media

**Figure 4:**
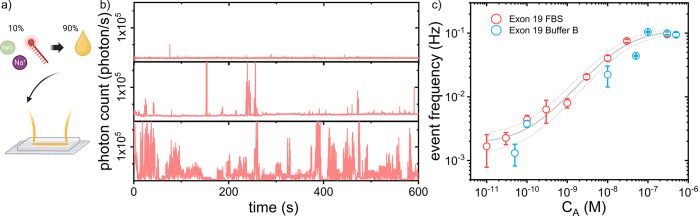
Sensor response for Exon 19 deletion in undiluted FBS. (a) Schematic of the sample preparation: the solution consisted of 10% of a 10-fold concentrated detection probe mixed with 90% serum, which was spiked with E19del. (b) Example timetraces of detection events on single nanorods in at analyte concentrations of 0 pM, 3 nM, and 30 nM respectively. (c) Dose–response curve for the detection of E19del in FBS (red circles) compared to E19del in sensing buffer (blue circles). Solid and dashed lines are fits according to eq 2. Fit parameters are Hill coefficient γ = 0.82, α = 2.04 × 10^–4^, and *k*_*on*_^(1)^/*k*_*off*_^(1)^ = 2.09 × 10^6^ M^–1^. The fit includes an offset of 1.8 × 10^–3^; the gray dotted lines indicate the uncertainty of the fit.

Next, we performed a biologically relevant assay for the analyte EGFR exon19 deletion (E19del). E19del is the most common activating mutation in advanced lung cancer and is widely used as a biomarker for choice of treatment.^42^ To gauge the performance of the PEF sensor in biological fluids, we performed these assays in unfiltered fetal bovine serum (FBS) by mixing FBS in a 9:1 ratio with a 10-fold concentrated detection probe. The solution was spiked with the analyte, and 150 mM sodium chloride and 1 μM dextran sulfate were added as depicted in Figure 4a. Following the previously established method, the capture and detection probes (see SI for their sequences) were redesigned to match the analyte, and the sensor response to different analyte concentrations was recorded.

As shown in Figure 4b, recorded TTs of individual particles show increasing activity in the presence of a higher analyte concentration with clear bursts. As expected, the superior signal-to-noise ratio of PEF enables single-molecule detection in serum without sample filtration or washing steps because the signal of specific binding events on the surface of the particle is orders of magnitude brighter than the serum background. Using an identical analysis method, the sensor responses in serum and in sensing buffer were compared as displayed in Figure 4c. We find similar dose–response curves as in buffer, indicating that the limit of detection is not compromised by the presence of serum proteins.

As can be seen in the direct fluorescence signal, the event durations and signal dynamics differ in serum from the assays in a clean buffer (see Figure 2 and Supplementary Figure S4). We hypothesize that a protein-rich environment affects the diffusion and capture of the analyte and detection probe, giving rise to different signal characteristics. Nevertheless, the recorded sensor response scales identically with analyte concentration in both controlled buffer and serum, hence demonstrating the compatibility of this technology with interference-rich matrices. In serum we find a higher LOD of 164 pM due to differences in the sequence design. Moreover, we hypothesize that interference-rich media gives rise to a higher degree of anticooperation in the formation of the DNA sandwich, which is evidenced by the lower average Hill coefficient γ = 0.76 across 3 repeats.

### Continuous Monitoring

The reversible sandwich assay enables affinity-based continuous monitoring. This enables the tracking of increasing and decreasing analyte concentrations over time, on time scales that are dictated by the affinity of the analyte for capture- and detection-probe as well as the fluidics. Regarding the latter, recent work has shown that the time to equilibrium can also be tuned by the channel height, but we keep this fixed at 400 μm for which confinement-induced changes in the time-to-equilibrium are negligible.^9,43^ We implemented the E19Del assay to demonstrate the ability to continuously monitor changes in the biomarker concentration. To improve the reversibility of the interactions, we reduced the complementarity between the analyte and capture probe from 11 to 7 nt. This shortens the bound-state lifetime of the analyte on the particle to approximately 1 s. The complementarity between the detection probe and the analyte is slightly longer in this design (9 nt), so the signal dynamics are largely dictated by the weakest interaction, namely, the one between the capture probe and analyte.

**Figure 5:**
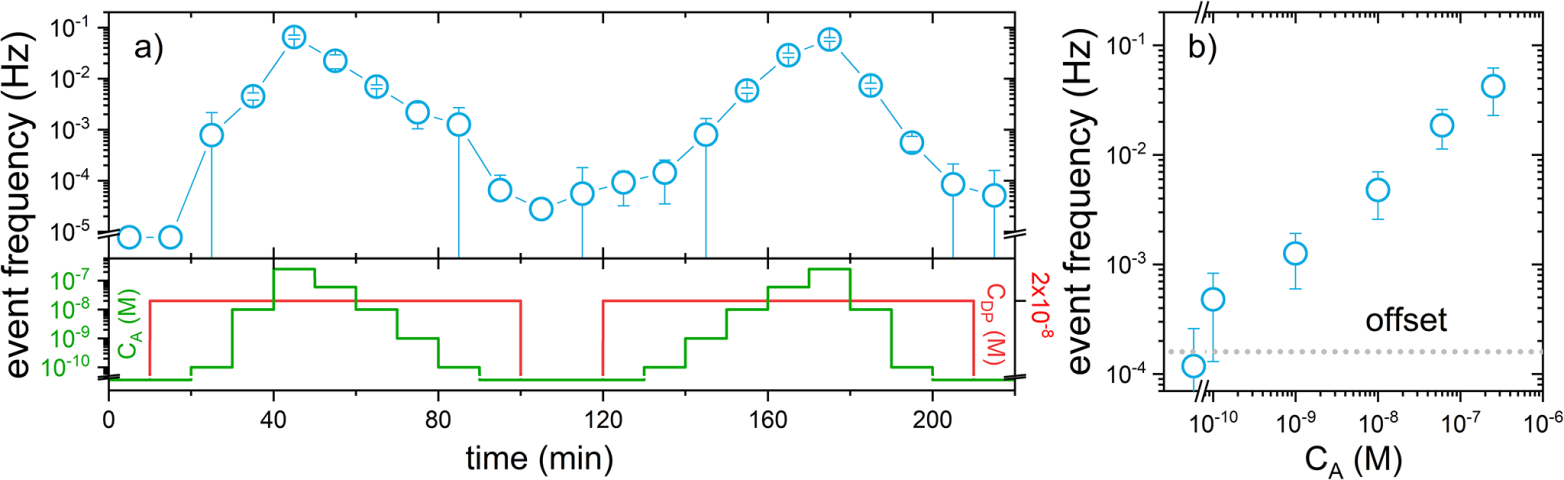
Continuous monitoring of E19del in sensing buffer. (a) Continuous monitoring over a duration of 3.5 h during which the concentration of E19del was sequentially increased and decreased at intervals of 10 min. Top panel: event frequency as a function of time. Bottom panel: step-like concentration changes from 100 pM to 250 nM (green), together with the detection probe concentration (red). (b) Average dose–response curve across 4 repeats of (a) on different sensor substrates, where the error bar indicates the standard deviation of all 10 concentration ramps across the 4 samples. The dashed line highlights the offset parameter obtained by fitting with eq 2: offset = 1.6 × 10^–4^.

Figure 5a shows the continuous monitoring of E19del over a period of approximately 3.5 h on the same chip. The concentration of the analyte was varied between 0 and 250 nM (see Figure 5a bottom panel), and the sensor’s activity was recorded. Continuous monitoring on this sensor does not require any washing or regeneration steps in between data points. Rather the fluid exchange in the flowcell was performed by simply injecting 1 mL of analyte-spiked buffer to mimic continuous concentration variations. Halfway through the concentration series, buffer solutions without analyte and detection probe were measured to gauge the background level. Interestingly, we observe no further reduction of the measured event frequency upon removal of the detection probe, indicating that plasmon-enhanced fluorescence of impurities in the buffer may be at the origin of the remaining events.

Both increasing and decreasing ramps were performed to probe the sensor’s temporal response to concentration changes. The sensor response closely tracks the analyte concentration with an integration time of 10 min. We observe an ∼10-fold lower event frequency compared to Figures 3 and 4 which is attributed to the increased dissociation constant *k*_*off*_^(1)^ due to the above-mentioned reduction of complementarity from 10 to 7 nt. This reduces the number of analytes bound per particle due to the reduced affinity. In order to partially compensate for the reduced event frequency, the detection probe concentration was doubled to 20 nM. This did not result in a noticeable reduction of the signal-to-noise ratio, indicating that plasmon enhancement effectively overcomes background from serum proteins and detection probes alike.

In Figure 5b, the average of the response of multiple sensors is plotted, in which these were exposed to the concentration series in Figure 5a. The sensor activity is highly reproducible across the 4 independently prepared sensors. For this assay, we find an LOD ≈ 1 nM with a Hill coefficient γ ≈ 0.9. This LOD is an interplay between the reduced event frequency due to the lower affinity between the analyte and capture probe, partially compensated by the increased concentration of the detection probe. The error bars in Figure 5b indicate the standard deviation of the mean from different substrates, highlighting robust batch-to-batch reproducibility. The sensor’s response exhibits a coefficient of variation of approximately 50% both in continuous sensing mode and complex media (SIFigure S5a). In the future, it would be interesting to improve the sensor-to-sensor reproducibility even further by incorporating reference particles that exhibit a known response to the detection probe.

## Conclusions

We demonstrated a biomolecular sensing technique based on the PEF on single biofunctionalized gold nanorods. PEF effectively overcomes background signals due to complex media such as serum, enabling single-molecule biosensing and digital event detection directly in undiluted biological fluids. By exploiting transient interactions with the analyte, we demonstrated the continuous monitoring of a cancer maker (E19del) with a low nanomolar LOD and temporal resolution of 10 min. The sensor is stable for more than 3 h, which can easily be extended substantially because the large sample surface allows for 10^4^–10^5^ measurements by interrogating a different (fresh) field-of-view.

In the future, the optical readout system can be miniaturized owing to the strong PEF that provides photon count rates in excess of 1 million photons per second. This enables miniaturization, because a low numerical aperture objective lens in combination with an affordable (uncooled) camera will still provide a sufficient signal-to-noise ratio. The modular design of this sensor allows adaptation to the detection of cytokines and other proteins using low-affinity probes such as aptamers,^44^ antibodies fragments,^45^ and peptides.^46,47^ Competition assays are also easily implemented by employing detection probes that directly bind (with low affinity) to the capture probe, thereby competing with the analyte. SM interactions also provide access to molecular dynamics information allowing for kinetic fingerprinting, hence improved specificity. Enhanced SM fluorescence sensing provides a stepping stone and cheap platform for continuous, multiplexed, and highly specific sensing directly in biofluids for monitoring in healthcare, supervision of industrial processes, and overseeing of ecological systems.

## Methods

### Sample Preparation of Biosensor Substrate

Borosilicate glass coverslips (thickness #1.5) (Menzel-Gläser 24 × 40 mm^2^) were sonicated in methanol for 15 min and dried by a gentle N_2_ flow. The glass substrates were activated by oxygen-plasma treatment for 1 min. Substrate modification was performed by incubating the slides in a solution of MPTMS (5% vol) in ethanol for 3 min, then rinsed with ethanol, and dried with a N_2_ flow. A suspension of AuNRs (A12-40-650-CTAB, NanoPartz) was centrifuged for 3 min at 10000 rpm and resuspended in 1 mM CTAB in Milli-Q water. The AuNRs were then spin-coated onto the coverslips, after which excess CTAB and unbound nanorods were removed by rinsing with methanol, phosphate-buffered saline (PBS), and distilled water. The immobilized rods were functionalized with thiolated single-strand DNA (IDT Technologies or Eurofins Genomics) where a solution containing 5 μM thiolated strands and 1 mM tris(2-carboxyethyl) phosphine hydrochloride (TCEP) in citrate buffer (100 mM, pH 3, 1 M NaCl) was drop-casted and incubated for 2–12 h on the slides following the protocol reported by the group of Liu.^48,49^ After functionalization, the samples were rinsed with PBS and sensing buffer (5 mM Tris-HCl, 10 mM MgCl_2_, 1 mM EDTA, pH 8.0, filtered). A flowcell (Grace Biolabs Custom SecureSeal RD478065 or iBidi sticky-Slide VI 0.4) was applied on a sensing substrate, and flow chambers were immediately filled with sensing buffer. Samples were stored for 2–10 days in a humidity chamber at 4 °C before measurement.

### Buffer Solutions

For biosensing in controlled buffered solutions, we used the above-mentioned sensing buffer (5 mM Tris-HCl, 10 mM MgCl_2_, 1 mM EDTA, pH 8.0, filtered) containing 10 nM detection probe labeled with ATTO655 (IDT Technologies or Eurofins Genomics). The solution containing the detection probe was spiked with the analyte at varying concentrations and immediately injected into the measuring chamber.

For experiments in complex media, we mixed a solution of fetal bovine serum (FBS) (Thermo Fischer Gibco 10082147) and 10× sensing buffer at a volume ratio of 9:1. The sensing buffer contained in addition 150 mM NaCl and 1 μM dextran sulfate (Sigma-Aldrich Calbiochem 265152-M). The mixture was vortexed and sonicated for 1 min, allowing the complete dissolution of dextran sulfate. After, the solution was spiked with a 10 nM detection probe labeled with ATTO655 and ssDNA analyte (IDT Technologies or Eurofins Genomics) at different concentrations. As before, the time that occurred between the mixing of the analyte and the detection probe and the measurement start was approximately 1 min. See the Supporting Information for sequence details.

### Optical Microscopy

Fluorescence image sequences were measured by objective-type total internal reflection microscopy on an inverted wide-field microscope (Nikon Ti2). A 637 nm fiber-coupled excitation laser (OBIS FP 637LX, Coherent), collimated by a Thorlabs F810APC-635, cleaned up (Thorlabs BP FLH635-10) and s-polarized, was used to illuminate the sample via a dichroic mirror (ZT640rdc, Chroma). The laser was focused (Thorlabs LA1172-A) on the back focal plane of an oil immersion objective (Nikon Apo TIRF 60x Oil DIC N2) and projected onto the sample with a power density of 2000 W·cm^–2^. In the detection path, the fluorescence signal is collected by the same objective, and the excitation light was further suppressed by a notch filter (ZET635NF, Chroma) and a long-pass filter (Thorlabs FELH0650). The fluorescence signal from the sample was focused via a tube lens (Thorlabs TTL200-A) on a Prime BSI Express Scientific sCMOS and recorded with an integration time of 100 ms. A typical field of view (FoV) is shown in Figure 1d, where each diffraction-limited spot is the result of the one-photon photoluminescence of single gold nanoparticles. Fluorescence data were analyzed using custom Python and Matlab software (see the Supporting Information for details).

### Dose–Response Curves

At the start of the experiments, the flowcell was filled with sensing buffer that contained only detection probes. The solution was replaced with flowing sensing buffer with increasing analyte concentrations. Solution exchange was performed by introducing 250 μL of the new solution via pipetting it into the inlet of the Grace Biolabs Custom SecureSeal flow cell (capacity approx 26 μL). Such volume and flow speed allow for the complete exchange of the solution in the measuring chamber.

### Continuous Monitoring Biosensing

Microscope slides 24 × 60 mm^2^ and iBidi sticky-Slide VI 0.4 were used that were connected to a pipet via L-shaped plastic adaptors and a short tube. Fluid exchange in the measuring chamber is performed by pipetting 1 mL of an analyte-spiked solution without intermediate washing steps. We use a pressure-induced flow produced by microfluidic pump to flow the sample over the sensor, although pump-free microfluidics could also be an interesting option.^50^ A sequence of concentrations was injeted every 10 min without pauses, resulting in a continuous modulation of concentration as shown in Figure 5.

### Numerical Simulation

Simulations of the optical properties of nanoparticles were carried out using the boundary element method (BEM), using the MNPBEM17 toolbox and following.^36^ The nanorod was modeled as a cylinder capped by hemispheres, with dimensions 40 nm by 82 nm resulting in a longitudinal plasmon at 640 nm. The dielectric function of gold was used as tabulated by Johnson and Christy,^51^ whereas the refractive index of the medium was set to 1.33. The substrate was neglected in the BEM simulations. The enhanced excitation rate was extracted by calculating the near-field intensity under plane-wave excitation at 637 nm with polarization along the long axis of the particle. The emission enhancement was extracted by calculating the enhancement of the radiative and nonradiative rates of a single-wavelength emitter at 680 nm. The emission enhancement was averaged over 3 dye orientations to simulate a freely rotating dye. The overall enhancement factor is then given by the product of excitation and emission enhancements, which is plotted in Figure 1f.

## Supporting Information Available

Materials and methods, DNA sequences, methods for the identification of single particles, extraction of time traces and event detection, single-molecule event filtering, further statistical analysis, the sensor analytical response parameters, additional E19del time traces in complex media, and a description of the model on binding kinetics. (PDF)
