# Supplementary material for: Continuous Monitoring Biosensing Mediated by Single-Molecule Plasmon-Enhanced Fluorescence in Complex Matrices
Source: ACS Nano. 2024 Feb 9;18(7):5805–13. doi: 10.1021/acsnano.3c12428 (PMC10883122; doi:10.1021/acsnano.3c12428)
Supplement: Supplementary file 1 — nn3c12428_si_001.pdf [file nn3c12428_si_001.pdf]

# Supplementary Information for: Continuous monitoring biosensing mediated by single-molecule plasmon-enhanced fluorescence in complex matrices

Vincenzo Lamberti, Mathias Dolci, and Peter Zijlstra*

Eindhoven University of Technology, Department of Applied Physics and Science Education and Institute for Complex Molecular Systems, 5600 MB Eindhoven, The Netherlands

* p.zijlstra@tue.nl, January 22, 2024

## Contents

**1 Sequences** **2**

**2 Identification of single particles** **2**

**3 Extraction of time traces and event detection** **4**

**4 Single-molecule event filtering** **4**

**5 Statistics of events per particle** **5**

**6 Sensor analytical response parameters** **5**

**7 EGFR Exon 19 deletion (E19del) in complex media** **7**

**8 Binding kinetics based on Langmuir kinetics** **8**

# 1 Sequences

| Name | Function | Sequence |
|---|---|---|
| **Assay: proof of concept 1.0** | | |
| A1.0$_{Dock}$ | Capture Probe | 5'- GTGATCGCTCGC ATAACCTTCG-CATACTACTAC - Thiol – 3' |
| A1.0$_{Analyte}$ | Analyte ssDNA | 5'- GCGAGCGATCAC AAA ATA-CATCTA – 3' |
| A1.0$_{imager}$ | Detection probe | 5'- CTAGATGTAT - ATTO655 – 3' |
| **Assay: EGFR Exon 19 deletion (E19del)** | | |
| A$Ex$19$_{Dock}$ | Capture probe | 5'- AGCGACGGGAA TTTTTTTTT - Thiol – 3' |
| A$Ex$19$_{RevDock}$ | Reversible capture probe | 5'- ACGGGAA TTTTTTTTT - Thiol – 3' |
| A$Ex$19$_{MAnalyte}$ | Mutant analyte ssDNA | 5'- TTCCCGTCGCT AT CAAGACATC TAGGAC – 3' |
| A$Ex$19$_{WTAnalyte}$ | Wild type analyte ssDNA | 5'- TTCCCGTCGCT AT CAAGGAATT-TAGGAC – 3' |
| A$Ex$19$_{imager}$ | Detection probe | 5'- CGATGTCTTG - ATTO655 – 3' |

**Table S1:** DNA sequences used in this work. Complementary bases are highlighted with the same color. Note that the detection probe is 10 nt long, however only 9 nucleotides contribute to the hybridization with the capture probe

# 2 Identification of single particles

To process only timetraces corresponding to single nanoparticles (rather than clusters), we characterized a proof-of-concept sandwich assay with correlative optical microscopy. We perform hyper-spectral scattering microscopy (HSM) to obtain scattering spectra of AuNR immobilized on glass and correlate with a typical sandwich assay fluorescence measurement. We exchange illumination and detection path on the same microscopy platform without interfering with the sample to acquire and correlate scattering and fluorescence data.
Both scattering and fluorescence image sequences were measured by objective-type total internal reflection microscopy on an inverted wide-field microscope (Nikon Ti2). In scattering, the sample was illuminated by a fiber-coupled white-light source equipped with a tunable band-pass filter (SuperK COMPACT and SuperK VARIA tunable filter). Collimated by a Thorlabs RC08FC-P01, excitation light is focused on the back focal plane of an oil immersion objective via a focusing lens (Thorlabs LA1172-A) and a beam splitter. Totally internal reflected beam is blocked by a custom-made beam-blocker located at the bottom of the objective. Scattered light is collected in the detection path without filters and focused on a Prime BSI Express Scientific sCMOS via tube lens (Thorlabs TTL200-A). Excitation light was tuned from 450$nm$ to 800$nm$ with bandwidth of 10$nm$ and set at 1% working power; for each step, the scattering intensity of the FoV was collected. Image sequences of 35 frames were analyzed using custom Python and Matlab software (see Analysis section) obtaining scattering spectrum of ROIs in the FoV. In order to perform fluorescence measurement, components were mounted on exchangeable supports allowing swapping without moving the sample and imaging the same FoV. Fluorescence illumination and detection elements are reported in the main text.

In Fig.S1.a and Fig.S1.b, we consistently observe that Lorentzian-shaped scattering spectra correlate to stable time traces with clearly defined burst peaks associated to analyte presence. On the contrary, high-intensity spots with multiple peaks and ambiguous spectra (Fig.S1.d) result in unstable time traces with either inconsistent levels, drifting baseline, or higher-than-usual intensity peaks (Fig.S1.e). We characterize these ROIs as clustered AuNR and manually discard them from further analysis. Approximately 5 to 10% of processed ROIs are discarded as clusters. Time traces with a stable baseline and no events are associated to single particles whereas no binding event takes place, but are accounted for in the statistical average of event frequencies.

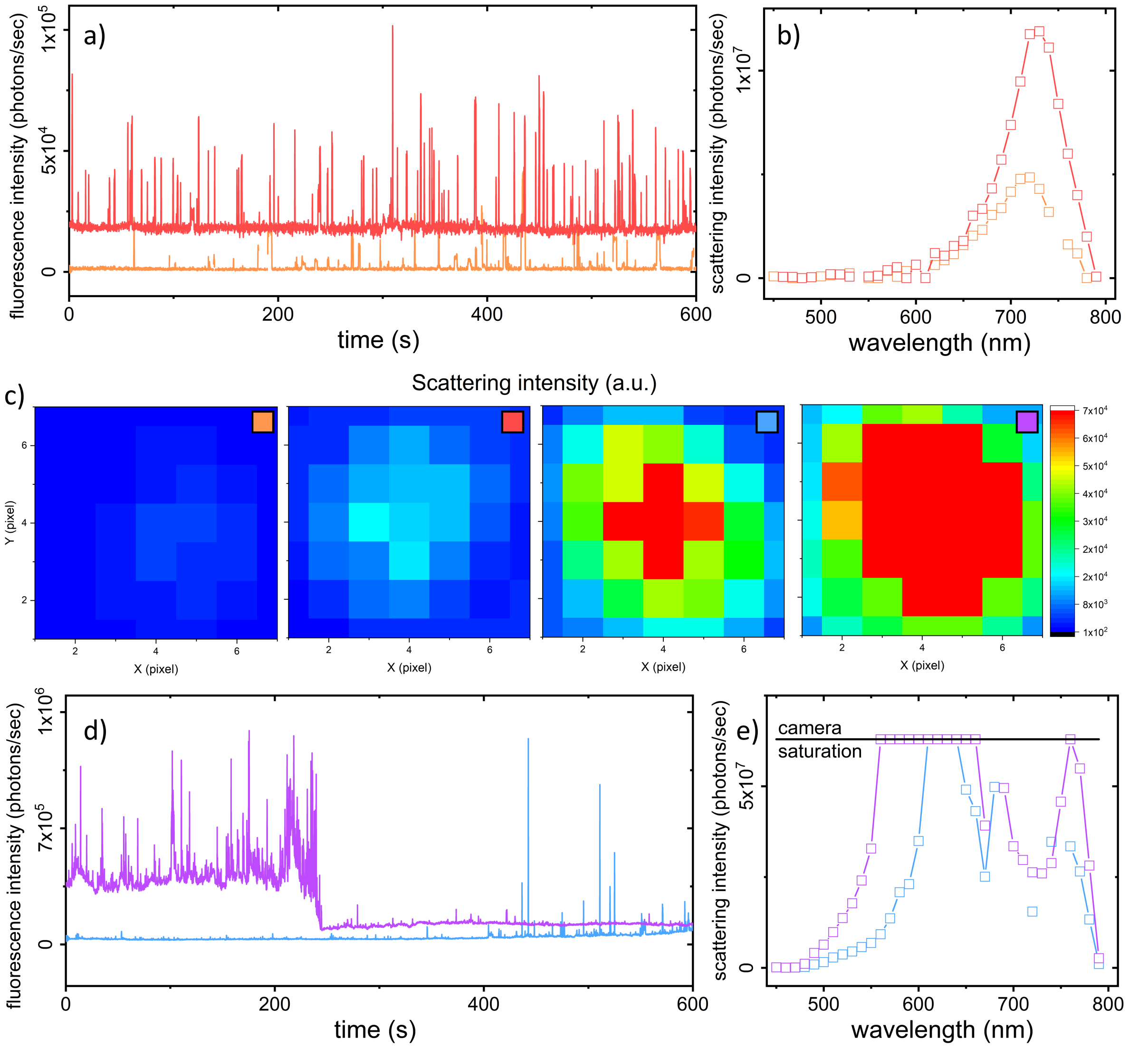


**Figure S1:** Typical time traces and HSM of ROIs. a) Time traces of two exemplar ROIs associated to single nanoparticles with stable baseline and clear peaks. b) Correlated scattering spectra of ROIs in panel a) color-coded per ROI. c) 2D intensity plot of ROIs at 650nm. Pixel size 0.11 $\mu$m. d) Time traces of two typical ROIs containing a particle clusters. Unpredictable behavior (magenta) or drifting baseline along with abnormally intense peaks (blue) are discarded. e) Correlated scattering spectra of ROIs in panel d). Both signals reach saturation and non-Lorentzian-shape spectra.

# 3 Extraction of time traces and event detection

From raw image sequences, we employ a custom Python-written software PLASMON to identify ROIs and generate individual time traces. Loaded data is processed with drift correction and highlights 9x9 pixel ROIs with higher intensity compared to the background. In order to resolve specific from nonspecific events happening very close to (but not on) the nanoparticle, we super-localize each frame for all ROI by fitting a 2D Gaussian with the Maximum Likelihood Method (MLE). For each ROI, a time trace is generated as the area under the fitted Gaussian for each frame. Such results are saved in a Matlab workspace format.
Within a custom Matlab app SPectrA, Time traces can be visualized and marked as cluster ROI. Within the app, we threshold time traces marking each event duration and dark time whereas the threshold is the maximum between method A and B. Threshold A is $\mu_A + 6\sigma$, where $\mu_A$ is the baseline estimated by the Hampel function and $\sigma$ is the standard deviation of the baseline. Threshold B is $3\mu_B$ as the moving minimum in a 400 frames window. Marked events per ROI are saved and processed by a Matlab script in order to perform kinetic fingerprint filtering and analysis of the average event frequency and fitting of bright and dark times per particle, as reported in the main text.

# 4 Single-molecule event filtering

We characterize each binding event by defining its duration (bright time), the time difference from the follow-up (dark time), and intensity. A more useful definition of intensity is defined as the unitless peak-to-background ratio (PBR). For each event, we consider the maximum intensity and divide by the local baseline intensity. Given this characterization, for each image acquisition, we remove events too short and with too small intensity. A rectangular filter in the PBR-bright time domain of size 3x0.1001 is applied removing events in the FoV with both intensity smaller than 3 times the baseline and duration shorter or equal to 1 frame ($100msec$). Blue-colored events in FigS2.a-S2.b are discarded from further analysis.

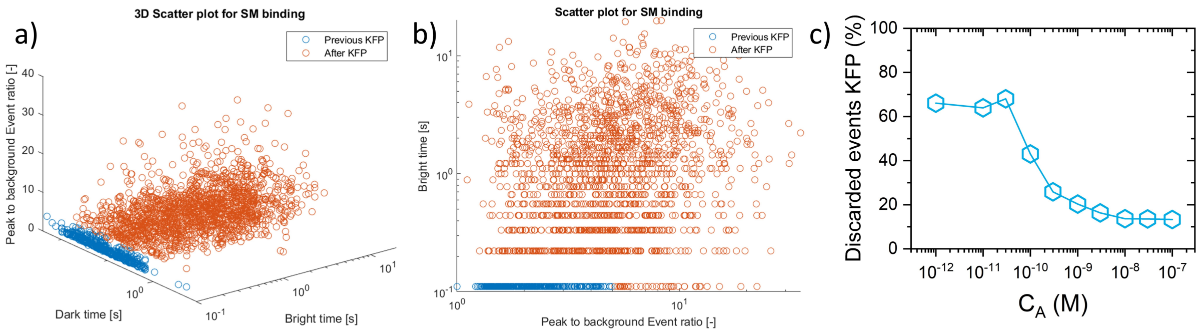


**Figure S2:** Typical event filtering report. Shown dataset: proof-of-concept assay 1.0 in controlled buffer (Buffer B) with $10nM$ detection probe and $1nM$ analyte. a) 3D scatter plot of all events characterized by PBR, bright time, and dark time. b) Scatter plot of all events characterized by PBR and bright time. c) Percentage of discarded events across DR.

## 5 Statistics of events per particle

Here, we report a typical distribution of the number of events per particle across different analyte concentrations. The mean of the distribution shifts and scales with concentration, while the width broadens as expected from a random distribution.

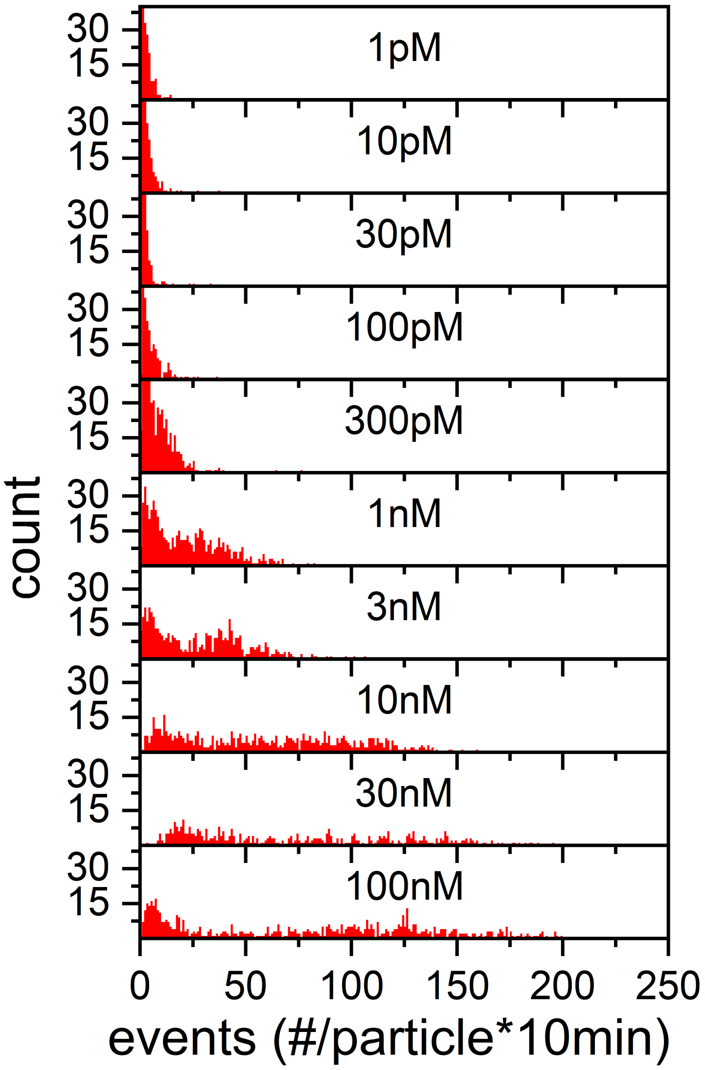


**Figure S3:** Histograms of single-particle event frequency across different analyte concentrations in controlled buffer (Buffer B) expressed as the number of events per particle per 10 minutes. Data from two sensor surfaces are accumulated for increased statistics. The bin size is fixed at 1 event/10min

## 6 Sensor analytical response parameters

We determine the sensor analytical parameters as LOD, LOQ, and EC50, as well as present the extended fit result of dose-response curves. Following the common approach[1], we determine the LOD and LOQ as follows:

$$LOD = \frac{3\,\sigma}{\partial f_E/\partial c_A}, \quad \text{and} \tag{S1}$$

$$LOQ = \frac{10\,\sigma}{\partial f_E / \partial c_A}, \tag{S2}$$

where the denominator indicates the sensor's sensitivity. The EC50 is identified by definition as the analyte concentration $c_A$ at which the event frequency $f_E$ is halfway between the minimum and maximum, yielding

$$EC50 = c_A^* \quad \text{such that} \tag{S3}$$

$$f_E(c_A^*) = \frac{max(f_E) - min(f_E)}{2}. \tag{S4}$$

We present these as averages and standard errors of 3-4 repeats of the same experiments.

| | EC50 | LOD | LOQ |
|---|---|---|---|
| Assay: proof of concept 1.0 | 5nM ± 2nM | 5pM ± 2pM | 20pM ± 6pM |
| Assay: Ex19del in complex media | 22nM ± 6nM | 160pM ± 18pM | 0.55nM ± 0.06nM |
| Assay: Ex19del Continuous detection | 60nM ± 12nM | 1.0nM ± 0.6nM | 4nM ± 2nM |

**Table S2:** Summary of the calculated analytical parameters

Similarly, we summarize the fit values from dose-response curves of the three different assays and conditions below. The static model system assay (assay 1.0) is designed such that the analyte-detection probe interaction is much weaker than the analyte-capture probe interaction. The measured bright times are then uniquely associated with the analyte-detection probe interaction. Here, we compare the measured averaged bright time of 1 second to values reported in the literature for the same sequence design in similar experimental conditions[2]. Using a similar sequence design approach, we designed the assay for the detection of Ex19del in complex media. Here the typical times differ from the model system; we attribute these differences to the following mechanisms: (1) Although the complementarity length of bases are the same (analyte-capture probe 12bp; analyte-detection probe 9bp) the higher GC content in these sequences would increase the bond lifetime, $\tau_b$, and (2) the complex environment in fetal bovine serum may have a strong influence on the affinity of DNA duplexes, either stabilizing or destabilizing the construct. We observe a stabilization and, hence, increasing fluorescence-burst duration experimentally. In the continuous detection assay, the bright times are slightly shorter due to the shorter complementarity of the sequences used.

The offset fit parameter is an indicator of the nonspecific interaction present in the sensor in the absence of both the analyte and detection probe, hence limiting the LOD of the sensor.

Lastly, the Hill coefficient $\gamma$ is related to the association behavior of the sandwich molecular complex. Specifically, a value of $\gamma$ smaller than 1 indicates a certain degree of anti-cooperativity in the formation of the capture-analyte-detection probe molecular complex. In other words, the binding if subsequent analyte or detection probe is hindered by the presence of already bound strands. The table below shows the extracted kinetic parameters as averages and standard deviations of 3-4 repeats of the same experiments on different sample surfaces.

| | $\tau_b$ [sec] | offset [Hz] | $\gamma$ |
|---|---|---|---|
| Assay: proof of concept 1.0 | 1.2 ± 0.4 | 2.3e-3 ± 2.2e-3 | 0.93 ± 0.10 |
| Assay: Ex19del in complex media | 1.9 ± 1.3 | 2.2e-3 ± 1.4e-3 | 0.76 ± 0.14 |
| Assay: Ex19del Continuous detection | 0.6 ± 0.4 | 1.6e-4 ± 1.1e-4 | 0.87 ± 0.14 |

**Table S3:** Summary of the calculated fitting parameters for dose-response curves

# 7 EGFR Exon 19 deletion (E19del) in complex media

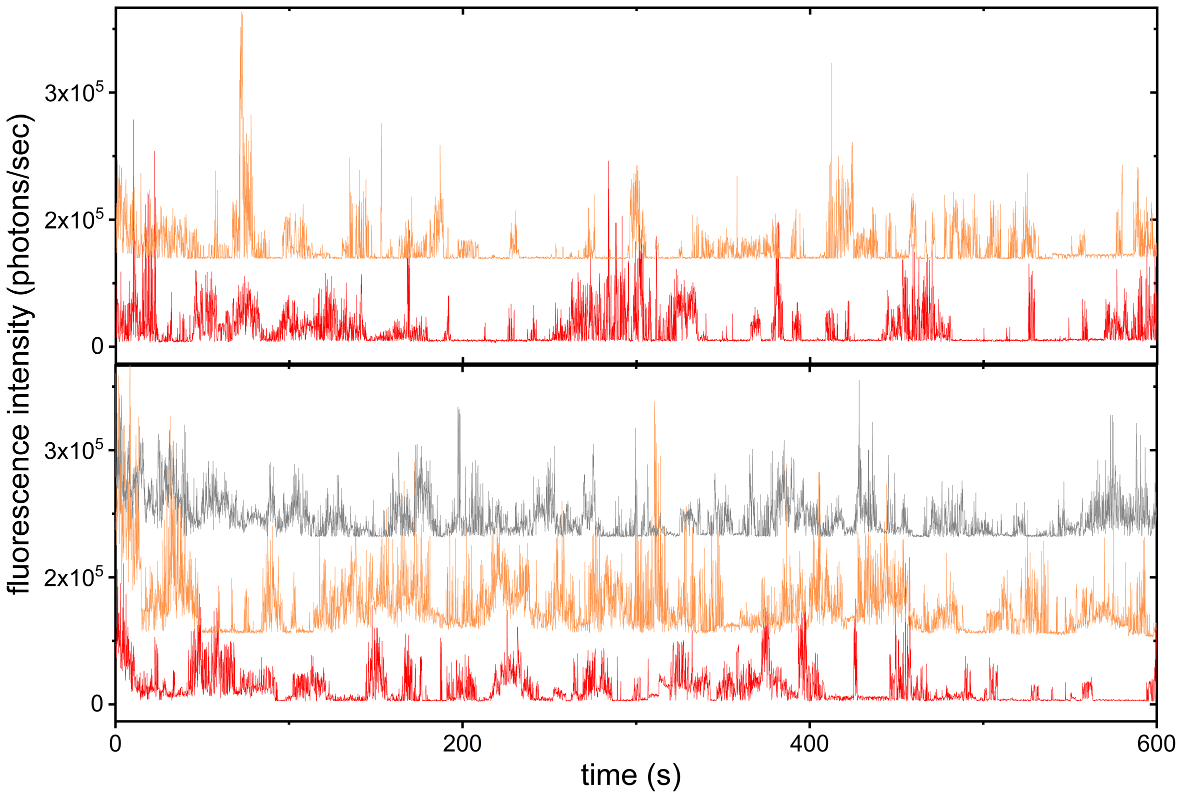


**Figure S4:** Exemplar time-traces for Exon19 assay in 90% Fetal Bovine Serum. Top: analyte concentration 30nM. Bottom: analyte concentration 300nM. Detection probe concentration is set at 10nM. Overall intensities have been shifted for visualization purposes.

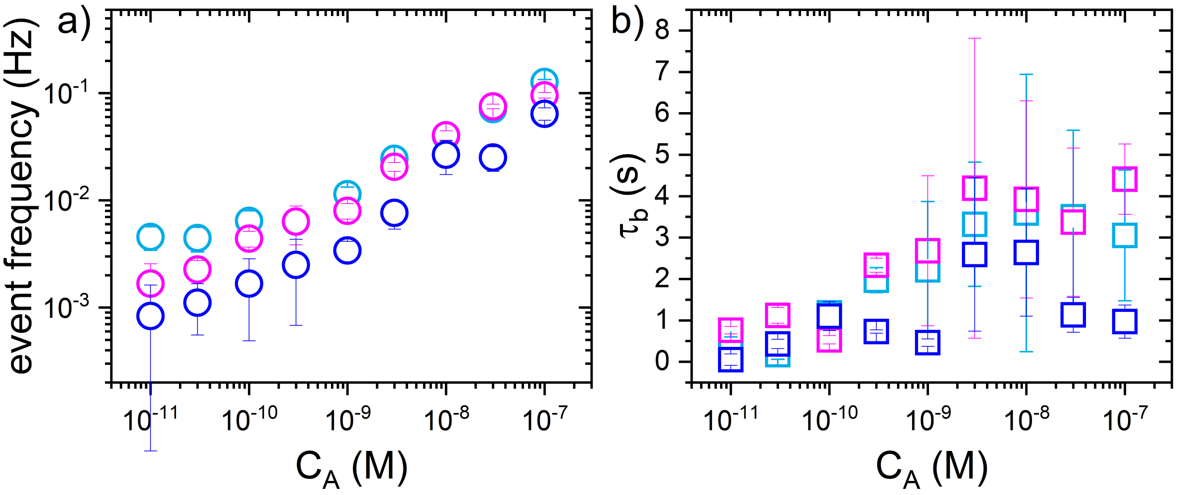


**Figure S5:** a) Replicates of DR curves of Exon 19 deletion in full FBS. Different curves are obtained from multiple substrates. b) Average $\tau_b$ across DRs.

# 8 Binding kinetics based on Langmuir kinetics

The binding frequency and therefore the number of events detected in a limited amount of time has multiple dependencies and can be derived from controllable quantities. Given the experimental conditions of target and detection probe concentrations, the number of events per particle and per time unit can be predicted using an adsorption model dictated by Langmuir kinetics. All the derived quantities have to be considered as results of a Poisson distributed process, therefore the obtained expressions are valid only as ensemble averages.

It's been experimentally proven that, for nanorods size of $40nm$ in diameter and $82nm$ length, the number of capture probes per particle is taken as $540 \pm 40$ (see main text).
The dose-response curve of the sensor is given by two time-separated dynamic interactions. In order to obtain an event, an analyte has to be bound to a capture probe, and successively, with the bond lifetime of the capture-analyte, a detection probe has to hybridize with the immobilized analyte. Consider the probability per unitary concentration that an analyte is bound to a capture probe on one particle.

$$p(CP-A) \propto \langle N_{available\ CP}\rangle_p \frac{k_{on}^{(1)}}{k_{off}^{(1)}} \qquad [1/M] \tag{S5}$$

This probability depends only on the equilibrium constant of the hybridization between capture and target. It is also proportional to the available number of capture probes per particle.
Given this probability, the average, dynamic number of analytes bound to a single particle is proportional to the analyte concentration $c_A$.

$$\langle N_A\rangle_{p,\bar{t}} = c_A \cdot p(CP-A) = c_A \langle N_{available\ CP}\rangle_p \frac{k_{on}^{(1)}}{k_{off}^{(1)}} \tag{S6}$$

Assuming only specific interaction between capture probe and target, the number of available capture probes decreases with increasing $c_A$ concentration.

$$\langle N_{availale\ CP}\rangle_p = \langle N_{CP}\rangle_p - \langle N_A\rangle_{p,\bar{t}}$$

Substituting this expression in Eq.S6, a form for the available number of capture probes can be found.

$$\langle N_{availale\ CP}\rangle_p = \frac{1}{1 + c_A \cdot k_{on}^{(1)}/k_{off}^{(1)}}\langle N_{CP}\rangle_p \tag{S7}$$

Therefore, we can solve Eq.S6 as follows.

$$\langle N_A\rangle_{p,\bar{t}} = \frac{c_A \cdot k_{on}^{(1)}/k_{off}^{(1)}}{1 + c_A \cdot k_{on}^{(1)}/k_{off}^{(1)}} \cdot \langle N_{CP}\rangle_p \tag{S8}$$

In this expression, the number of analytes per particle is considered to be time averaged since multiple targets can bind and unbind to the multiple capture probes of a single particle. The time average is taken in a frame much larger than the bond lifetime between capture probe and target:

$$\bar{t} \gg \tau_{CP-A}^{lifetime} = 1/k_{off}^{(1)}$$

Given the information in TabS1 and experimental observations, $\tau_{CP-A}$ ranges with 1 to 10 seconds. Therefore, an observation time of 10 minutes is assumed sufficient to estimate this time average $\langle N_A\rangle_{p,\bar{t}}$. Within experimental timescales bigger than 100 seconds, it can be assumed that the number of targets per particle is only dependent on the analyte concentration.

Within these assumptions, it's also necessary to identify an upper limit for the number of analytes per particle.

$$\langle N_A\rangle_p \leqslant \langle N_{CP}\rangle_p = 540 \tag{S9}$$

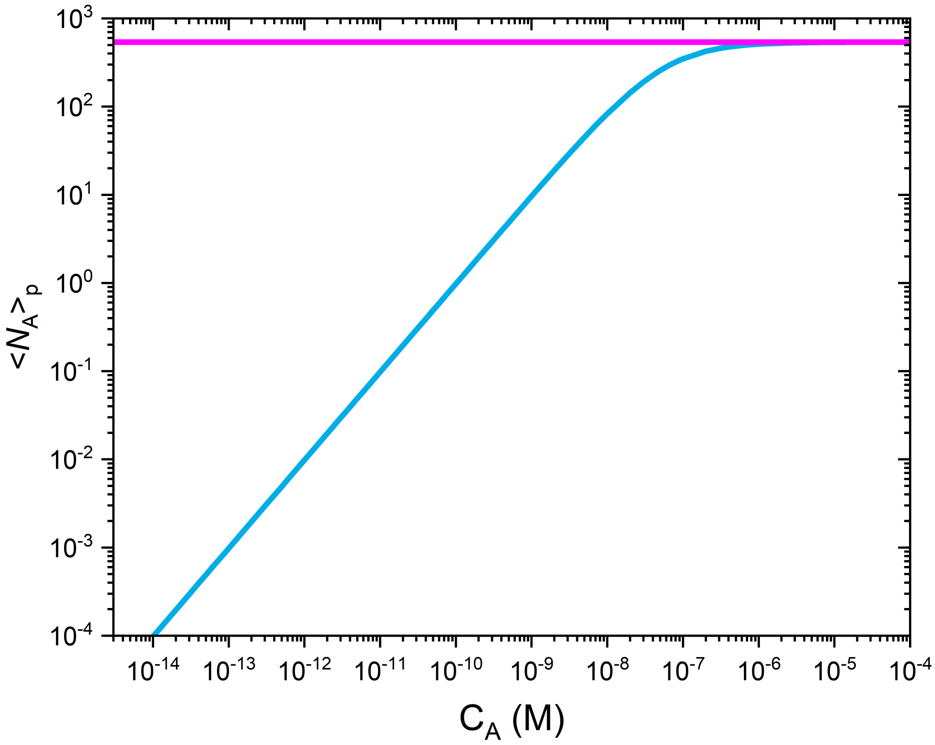


**Figure S6:** Relation between $\langle N_A \rangle_p$ and $C_A$. The Magenta line identifies the upper limit for the sensor, given the particle size considered, of 540 average analyte per particle, one on each available capture probe.

Given Eq.S8 and known the detection probe concentration $c_{DP}$, it is possible to predict the event frequency and therefore the average proprieties of the experimental fluorescence time trace. In a typical experimental setting, the binding kinetics of the sandwich assay is recorded for 10 minutes allowing to generate fluorescence time traces of 600 seconds. Considering the association rate $k_{on}^{(2)}$ between analyte and detection probe, the binding frequency of the florescence probe can be written as follow.

$$\langle f_E \rangle_p = c_{DP} \cdot k_{on}^{(2)} \cdot \langle N_{available\ A} \rangle_p \tag{S10}$$

Eq.S10 is expressed as number of events per second, $[\#/s]$.

Similarly to Eq.S7, the available number of bounded analytes can rewritten as a function of the detection probe concentration.

$$\langle N_{available\ A} \rangle_p = \frac{1}{1 + c_{DP} \cdot k_{on}^{(2)}/k_{off}^{(2)}} \cdot \langle N_A \rangle_p \tag{S11}$$

Combining Eq.S11 and Eq.S8 in Eq.S10, it is possible to predict the event frequency of the assay as a function of $c_A$ and $c_{DP}$.

$$\langle f_E \rangle_p = \frac{c_{DP} \cdot k_{on}^{(2)}}{1 + c_{DP} \cdot k_{on}^{(2)}/k_{off}^{(2)}} \cdot \frac{c_A \cdot k_{on}^{(1)}/k_{off}^{(1)}}{1 + c_A \cdot k_{on}^{(1)}/k_{off}^{(1)}} \cdot \langle N_{CP} \rangle_p \tag{S12}$$

Within the experimental setup, multiple events can overlap in fluorescence time trace, leading to a misinterpretation in event counting. To avoid this effect, a limit on event

frequency is imposed. Event under-counting can be prevented if events are distantly spaced in time and, therefore not too frequent. Here, we set a limit in terms of event frequency of $0.3 Hz$. In a typical acquisition time of 600 seconds, this corresponds to a maximum number of events per particle per dataset of 200. The observed number of event within an experimental dataset can be predicted multiplying Eq.S12 by 600 seconds, the acquisition time. Here we consider a fixed detection probe concentration of $10 nM$.

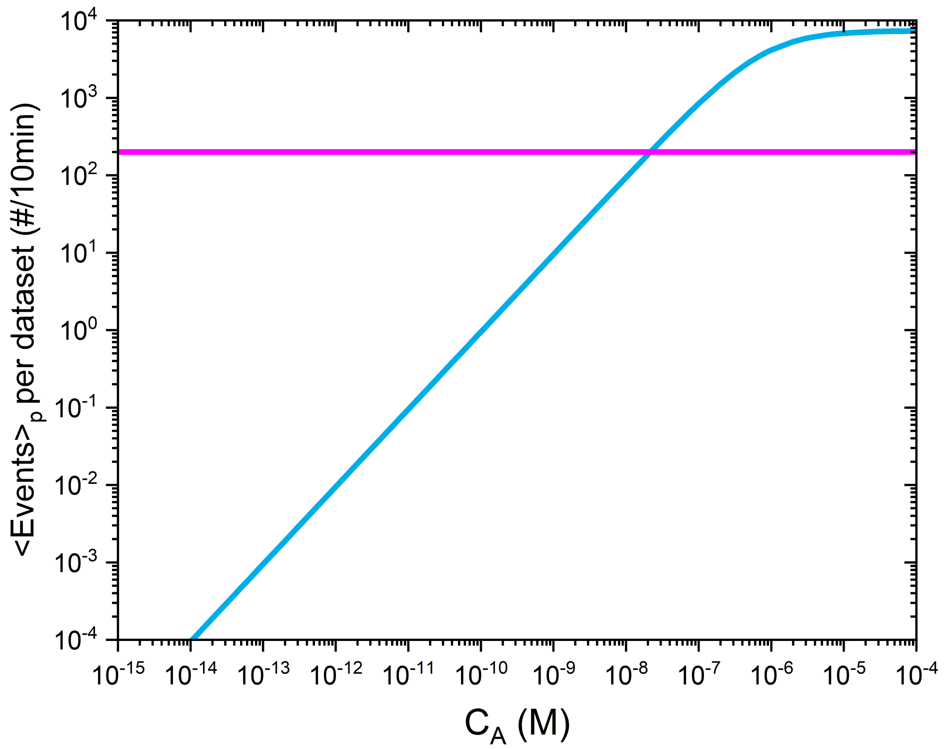


**Figure S7:** Relation between $\langle Events \rangle_p$ per 10 min and $c_A$. In this graph, a concentration of 10 nM detection probe is considered. The Magenta line identifies the upper limit for the sensor, considering a maximum event frequency of 0.3 Hz.

# References


(1) Voigtman, E., *Limits of detection in chemical analysis*; John Wiley & Sons: 2017.

(2) Horáček, M.; Engels, D. J.; Zijlstra, P. Dynamic single-molecule counting for the quantification and optimization of nanoparticle functionalization protocols. *Nanoscale* **2020**, *12*, 4128–4136.
